# Soluble Isoform of the Receptor for Advanced Glycation End Products as a Biomarker for Postoperative Respiratory Failure after Cardiac Surgery

**DOI:** 10.1371/journal.pone.0070200

**Published:** 2013-07-23

**Authors:** Tokujiro Uchida, Nagara Ohno, Miho Asahara, Yoshitsugu Yamada, Osamu Yamaguchi, Makoto Tomita, Koshi Makita

**Affiliations:** 1 Department of Anesthesiology, Tokyo Medical and Dental University, Graduate School of Medicine, Tokyo, Japan; 2 Department of Anesthesiology, The University of Tokyo, Graduate School of Medicine, Tokyo, Japan; 3 Department of Critical Care Medicine, Yokohama City University Medical Center, Kanagawa, Japan; 4 Clinical Research Center, Tokyo Medical and Dental University Hospital of Medicine, Tokyo, Japan; University of Miami, United States of America

## Abstract

**Purpose:**

Postoperative respiratory failure is a major problem which can prolong the stay in the intensive care unit in patients undergoing cardiac surgery. We measured the serum levels of the soluble isoform of the receptor for advanced glycation end products (sRAGE), and we studied its association with postoperative respiratory failure.

**Methods:**

Eighty-seven patients undergoing elective cardiac surgery were enrolled in this multicenter observational study in three university hospitals. Serum biomarker levels were measured perioperatively, and clinical data were collected for 7 days postoperatively. The duration of mechanical ventilation was studied for 28 days.

**Results:**

Serum levels of sRAGE elevated immediately after surgery (median, 1751 pg/mL; interquartile range (IQR) 1080–3034 pg/mL) compared with the level after anesthetic induction (median, 884 pg/mL; IQR, 568–1462 pg/mL). Postoperative sRAGE levels in patients undergoing off-pump coronary artery bypass grafting (median, 1193 pg/mL; IQR 737–1869 pg/mL) were significantly lower than in patients undergoing aortic surgery (median, 1883 pg/mL; IQR, 1406–4456 pg/mL; *p* = 0.0024) and valve surgery (median, 2302 pg/mL; IQR, 1447–3585 pg/mL; *p* = 0.0005), and postoperative sRAGE correlated moderately with duration of cardiopulmonary bypass (*r_s_* = 0.44, *p*<0.0001). Receiver operating characteristic curve analysis demonstrated that postoperative sRAGE had a predictive performance with area under the curve of 0.81 (95% confidence interval 0.71–0.88) for postoperative respiratory failure, defined as prolonged mechanical ventilation >3 days. The optimum cutoff value for prediction of respiratory failure was 3656 pg/mL, with sensitivity and specificity of 62% and 91%, respectively.

**Conclusions:**

Serum sRAGE levels elevated immediately after cardiac surgery, and the range of elevation was associated with the morbidity of postoperative respiratory failure. Early postoperative sRAGE levels appear to be linked to cardiopulmonary bypass, and may have predictive performance for postoperative respiratory failure; however, large-scale validation studies are needed.

## Introduction

Respiratory failure is a relatively common postsurgical complication, which can prolong the duration of stay in the intensive care unit (ICU) in patients undergoing cardiac surgery [1], [2]. The reported incidence of respiratory failure ranges from 3–20%, depending on the definition of this complication [3]–[5]. Several risk factors for respiratory failure after cardiac surgery have been analyzed previously [6], [7]; however, most were related to demographic information or early primary myocardial dysfunction and hemodynamic instability [8], [9]. Currently, evaluation for postoperative lung condition is limited to ventilatory parameters, arterial blood gas analysis and a radiographic approach. These methods are most common in clinical practice; however, they might not reflect the degree of damage to the lung tissue immediately after surgery. In this context, biomarkers reflecting lung tissue damage might be helpful for quantification of the severity of lung injury. Furthermore, early prediction for high-risk patients for development of respiratory failure enables early initiation of effective therapy and preventive intervention. In this context, plasma biomarkers may have potential to improve predicting performance.

The receptor for advanced glycation end products (RAGE) is a cell surface protein which is abundantly expressed in the lung [10] and its expression has been demonstrated on the basal membranes of alveolar type I epithelial cells [11]. We previously reported that the soluble isotype of RAGE (sRAGE) can be used as a biomarker of alveolar epithelial injury in an experimental study and in patients with acute lung injury [12]. Subsequent studies also found that plasma sRAGE levels were associated with short-term outcomes in patients undergoing lung transplantation [13]–[15]. Therefore, the principal objective of the present study was to determine whether early postoperative levels of sRAGE could be associated with the morbidity of respiratory failure after cardiac surgery. We also studied whether early postoperative elevation of serum sRAGE is related with the severity of impairment of oxygenation and radiographic appearance.

Because several recent reports suggested that extrapulmonary inflammation [16], [17] could influence the serum levels of sRAGE, we studied whether we could eliminate the contribution of systemic inflammation (including endothelial injury) to the postoperative serum levels of sRAGE in our study cohort. For this purpose, we analyzed postoperative levels of interleukin-8 (as a biomarker for systemic inflammation) and von Willebrand factor antigen (vWF, as a biomarker for endothelial injury) concurrently with sRAGE, and studied the correlations between these biomarkers.

## Methods

This study was designed as a multi-center study organized by the Japan Society of Respiratory Care Medicine. Three university hospitals participated in this study, and approval for the protocol was obtained from each institutional review board for clinical studies (Clinical Research Center, Tokyo Medical and Dental University Hospital, Faculty of Medicine; Research Ethics Committee of The University of Tokyo Hospital; and General Administration Division, General Affairs Section, Yokohama City University Medical Center). Each patient provided written informed consent. This study is registered in the UMIN Clinical Trials Registry (ID: UMIN000010674; www.umin.ac.jp/ctr/index/htm).

### Patients

Between 4 February 2008 and 6 July 2009, patients undergoing cardiac surgery in each of the three institutions participated in this study. Preoperative exclusion criteria were: 1) patient’s age <20 years; 2) history of myocardial infarction within 30 days before the day of surgery; 3) preoperative diagnosis of pneumothorax; 4) diagnosis of chronic obstructive pulmonary disease; 5) neuromuscular disease; 6) morbidly obese (body mass index >40); and 7) systemic connective tissue disease. Postoperative exclusion criteria were: 1) severe neurological complications requiring mechanical ventilation; 2) the need for an extracorporeal circuit due to cardiac failure; and 3) patients whose pulmonary artery occlusion pressure (PAOP) was >18 mm Hg in the first postoperative week.

### Protocol for Postoperative Mechanical Ventilation

Postoperative mechanical ventilation in the ICU began with synchronized intermittent mandatory ventilation (SIMV) with pressure support (PS) (5–10 cm H_2_O) with the same ventilator setting used in the operating room. We aimed to decrease the frequency of mandatory ventilation to the range necessary to maintain minimal minute volume (>30 mL/kg of body weight), and then decreased the PS level if needed. PEEP and F_I_O_2_ were set according to the patient’s oxygenation level (PaO_2_/F_I_O_2_). First, F_I_O_2_ was reduced stepwise to 0.4 then PEEP was decreased stepwise in maximal increments of 3 cm H_2_O to the final target value of 5 cm H_2_O.

A weaning trial was attempted at least three times a day, and the result was judged successful when a patient passed the following tests successfully: 1) frequency of spontaneous breathing (f_spont_) <25 breaths/min; 2) maintenance of arterial partial pressure of carbon dioxide (PaCO_2_) between 35 and 50 mm Hg; 3) SpO_2_>90%; 4) patient remained clinically stable; and 5) in cases without mandatory ventilation, tidal volume >5 ml/kg of predicted body weight and rapid shallow breathing index (RSBI, calculated as f_spont_/tidal volume (L)) <100 within the allowed pressure support range. Predicted body weight (PBW) was calculated from the following equation: male PBW (kg) = 50+0.91(height (cm) −152.4), and female PBW (kg) = 45.5+0.91(height (cm) −152.4).

During the weaning process, a change to controlled ventilation was conducted in cases of induction of general anesthesia, e.g., for an invasive procedure or surgery.

### Biomarker Assays

For the measurements of plasma biomarker levels, blood was sampled: 1) immediately after the induction of general anesthesia (Baseline); 2) at admission to ICU immediately after the operation (Post-Op); and 3) the day after the operation (POD 1). After the collection of arterial blood in serum separation tubes and citrated plasma sampling tubes, samples were centrifuged at 3000 g for 15 min at 4°C. Serum and plasma were separated and stored at −80°C in each institution until biomarker measurements were performed. Once all the samples were collected, they were transported to one institution (T.M.D.U.), and sRAGE, interleukin-8, and vWF were measured by one physician who was blinded to the patients’ clinical data, using commercially available ELISA kits (Quantikine DRG00, R&D Systems, Minneapolis, MN for RAGE analysis; Quantikine D8000C, R&D Systems, Minneapolis, MN for interleukin-8 analysis; and IMUBIND vWF ELISA, American Diagnostica Inc., Stanford, CT for vWF antigen analysis). Each measurement was performed in duplicate following the manufacturer’s instructions. vWF antigen levels are expressed as a percentage of a normal pooled plasma control reference that was assayed against a secondary standard of the 4th International Standard for vWF [18]. Serum levels of NT-proBNP were measured by a clinical laboratory (SRL Inc., Tokyo, Japan).

### Clinical Data Recordings

Clinical data were collected from the medical records. Chest radiographs were taken during the first admission to ICU (POD 0), POD 1, 3, and 7, and scored by the number of quadrants of alveolar consolidation [19]. Chest radiographs were quantified by an attending physician blinded to the values of the biomarkers. The worst score among the four chest radiographs was recorded. Respiratory failure was defined according to the New York State Department of Health (NYSDH) registry as: pulmonary insufficiency requiring intubation and ventilation for >72 h postoperatively [3], [6], [9].

### Statistical Analyses

Total study size was designed using PS Power and Sample Calculations version 3.0 (http://biostat.mc.vanderbilt.edu/PowerSampleSize). Based on the data in the pilot study performed by the manufacturer of the ELISA kit, the standard deviation of serum levels of sRAGE was 755 pg/mL. We assumed the frequency of postoperative respiratory failure at 10% with the true difference of means between patients with or without respiratory failure of 1000 pg/mL. To reject the null hypothesis that the population means of these two groups are equal with statistical power of 0.9, we needed at least 7 patients in the respiratory failure group and 63 patients without respiratory failure.

All other statistical analyses were performed using STATA/IC software (version 11, StataCorp, College Station, TX, USA). For two-group comparisons, we used the Mann-Whitney U-test. For intergroup comparisons, we used the chi-square test for categorical data, and analysis of variance with Tukey’s test was used for post-hoc multiple comparisons for continuous numerical data. The biomarker levels were natural log-transformed for linearity. To find clinical variables with significant association with Post-Op sRAGE, multiple regression analysis was performed. Clinical data consisted of patients’ demographic data (age, gender, body weight, and smoking history); past history (diabetes mellitus); the laboratory data at admission to the ICU (white blood cell count, hemoglobin, platelet count, albumin, creatinine, total bilirubin, Post-Op PaO_2_/F_I_O_2_, PEEP levels at ICU admission, and APACHE II score at admission to ICU); and the variables from surgery (intraoperative packed red cell transfusion, duration of operation and cardiopulmonary bypass (CPB), PEEP levels at ICU admission, intraoperative tidal volume). Because the results of this multiple regression analysis showed that Post-Op sRAGE levels significantly correlated with duration of operation, duration of CPB, and APACHE II score, we analyzed the association between Post-Op sRAGE levels and morbidity of prolonged ventilatory support (>3 days) by multiple logistic regression analyses, using these three variables as possible confounding factors. We used Spearman’s test for correlation analyses and the correlation was graded depending on the r_s_ value: 0.7> *r_s_* >0.4 as moderate correlation; and 0.4> *r_s_* >0.2 as weak correlation. To evaluate the predictive performance for postoperative prolonged ventilatory support (>3 days), non-parametric receiver operating characteristic (ROC) curves were computed. The area under the curve (AUC) was calculated with a 95% confidence interval and chi-square tests were performed for comparisons. The optimal cut-off point was determined by calculating Youden’s index [20], [21]. Statistical significance was defined as *p*<0.05.

## Results

**Figure 1:**
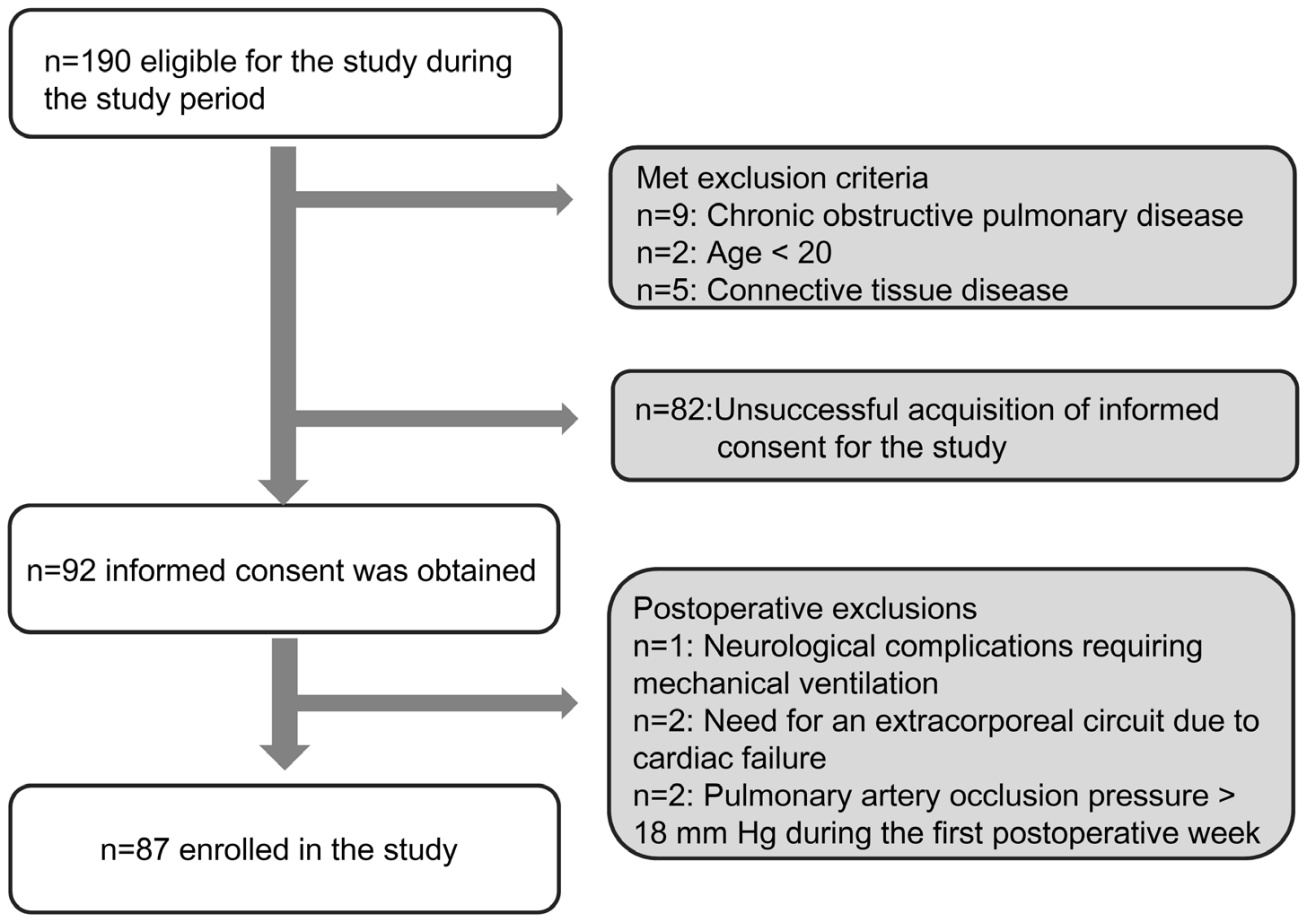
Flow chart for the enrollment of patients.

Between 4 February 2008 and 6 July 2009, 190 patients undergoing elective cardiac surgery were eligible for this study (100 patients in The University of Tokyo between 4 February 2008 and 26 September 2008; 49 patients in the Tokyo Medical and Dental University between 6 February 2009 and 22 June 2009; and 41 patients in the Yokohama City University Medical Center between 8 May 2009 and 6 July 2009). Informed consent was obtained from 92 patients for this study (32 patients in The University of Tokyo; 33 patients in the Tokyo Medical and Dental University; and 27 patients in the Yokohama City University Medical Center). After the analysis of the results from the first institution (n = 32, The University of Tokyo), we added two institutions to reach the required cohort size. One patient was withdrawn because of severe brain infarction diagnosed on POD 1 and the need for prolonged mechanical ventilation without lung complications. Two patients were withdrawn because of the need for percutaneous cardiopulmonary support on POD 3 due to cardiac failure. PAOP was monitored in 64 patients, and two were excluded because the PAOP exceeded 18 mm Hg during the first postoperative week. Postoperative levels of NT-proBNP in patients without PAOP monitoring (n = 25; median, 107 pg/mL; interquartile range (IQR), 69–289 pg/mL) were significantly lower than those managed with PAOP monitoring (n = 62; median, 233 pg/mL; IQR 91–871 pg/mL; *p = *0.016). As a result, eighty-seven patients were included in this study, and this process is summarized in Figure 1.

The baseline demographic data and clinical variables are summarized in Table 1. Twenty-one patients underwent aortic surgery, 33 patients underwent valve surgery, and 33 patients underwent off-pump coronary artery bypass grafting (OPCAB). The duration of operation was significantly longer in the aortic surgery group (*p* = 0.0002, vs. the valve surgery group; *p*<0.0001, vs. the OPCAB group), whereas there were no significant differences in the CPB time between the aortic surgery group and the valve surgery group. Intraoperative tidal volume was 9.3±1.4 mL/kg, and there were no significant difference among three surgical groups. Although intraoperative PEEP levels were slightly higher in the aortic surgery group, there were no significant differences in peak inspiratory pressure among the three surgical groups. In patients whose PAOP was monitored (n = 62), there were no differences in PAOP among the three surgical groups (10±4 mmHg in the aortic surgery group (n = 5); 12±3 mmHg in the valve surgery group (n = 32); and 10±4 mmHg in the OPCAB group (n = 25), *p = *0.11). More transfusions were performed in the aortic surgery group compared with the other two groups (Table 1). One patient (1.1%) died on POD 24 and another (1.1%) on POD 57, and respiratory failure requiring intubation and ventilation for at least 72 h postoperatively was observed in twelve patients (14.9%; seven in the aortic surgery group, three in the valve surgery group, and two in the OPCAB group).

**Table 1:** Patient characteristics.

	Total	Aortic Surgery	Valve surgery	OPCAB	p-value
Number of patients	87	21	33	33	
***Baseline characteristics***					**ANOVA**
Age (year)	64.0±12.0	60±13	63±14	68±8#	0.04*
Body weight (kg)	61.4±11.7	63.9±10.7	59.1±13.1	62.2±10.6	0.30
White blood cell count (cells/mm^3^)	5918±1549	6015±1470	5779±1532	5992±1648	0.81
Hemoglobin (g/dL)	12.5±1.6	12.3±1.3	12.5±1.3	12.8±1.9	0.53
Platelet count (10^4^/mm^3^)	20.2±6.3	19.2±7.0	19.6±5.5	21.4±6.4	0.36
Total protein (g/dL)	7.1±0.5	7.0±0.5	7.0±0.5	7.2±0.6	0.47
Albumin (g/dL)	4.1±0.5	4.1±0.4	4.2±0.4	4.0±0.4	0.25
BUN (mg/dL)	19.8±10.3	17.6±7.0	19.2±6.9	21.8±14.0	0.32
Creatinine (mg/dL)	1.1±0.7	1.2±0.9	1.0±0.3	1.2±0.8	0.37
Mean Blood Pressure (mm Hg)	91±13	93±15	85±11$$	95±11	0.004*
Heart rate (beats/min)	71±12	70±11	72±13	70±13	0.71
PaO_2_/F_I_O_2_ after the anesthetic induction	412±112	394±116	427±110	408±112	0.56
					**Chi square test**
Male/Female, (Male %)	64/23 (74%)	16/5 (76%)	21/12 (64%)	27/6 (82%)	0.68
Diabetes Mellitus +/−, (Morbidity %)	27/60 (31%)	1/20 (4.8%)	9/24 (27%)	18/15 (56%)	0.006**
Hemodialysis +/−, (Morbidity %)	2/85 (2.3%)	1/20 (4.8%)	1/32 (3.0%)	0/33 (0%)	0.50
History of smoking +/−, (Morbidity %)	51/36 (59%)	16/5 (76%)	20/13 (61%)	16/17 (48%)	0.44
***Surgical duration***					**ANOVA**
Duration of operation (min)	400±130	515±166	384±80^##^	343±99^##^	<0.0001
					**Mann-Whitney U test**
Duration of cardiopulmonary bypass (min)		213±109	197±52		0.33
***Intraoperative mechanical ventilation***					**ANOVA**
Tidal volume (mL/kg of PBW§1)	9.3±1.4	9.3±1.3	9.5±1.4	9.1±1.3	0.52
Peak inspiratory pressure (cmH_2_O)	20±5	21±5	20±5	18±5	0.12
PEEP level (cmH_2_O)	5±2	6±2$$	5±1	5±1	0.006**
***Intraoperative transfusion***					**Kruskal-Wallis test**
Red cell (units§2) §4		4 (0–8)	0 (0–4)	0 (0–2)#	0.0097**
Fresh frozen plasma (units§2) §4		6 (0–12)	0 (0–6)#	0 (0–0)^##^	0.001**
Platelet(units§3)§4		20 (0–20)	0 (0–0)^##^	0 (0–0)^##^	<0.0001**

OPCAB: off-pump coronary artery bypass grafting.

# *p*<0.05 vs. aortic surgery group.

* *p*<0.05.

$$ *p*<0.01 vs. OPCAB group.

** *p*<0.01.

§1 PBW: predicted body weight; male PBW (kg) = 50+0.91(height (cm) –152.4), and female PBW (kg) = 45.5+0.91(height (cm) –152.4).

§2 1 unit of packed red cells or fresh frozen plasma is derived from 200 mL of donated blood.

§3 1 unit of packed platelet concentrate contains 2×10^10^ platelets.

§4 Data are shown as median with interquartile range.

### Perioperative Change in the Levels of sRAGE

**Figure 2:**
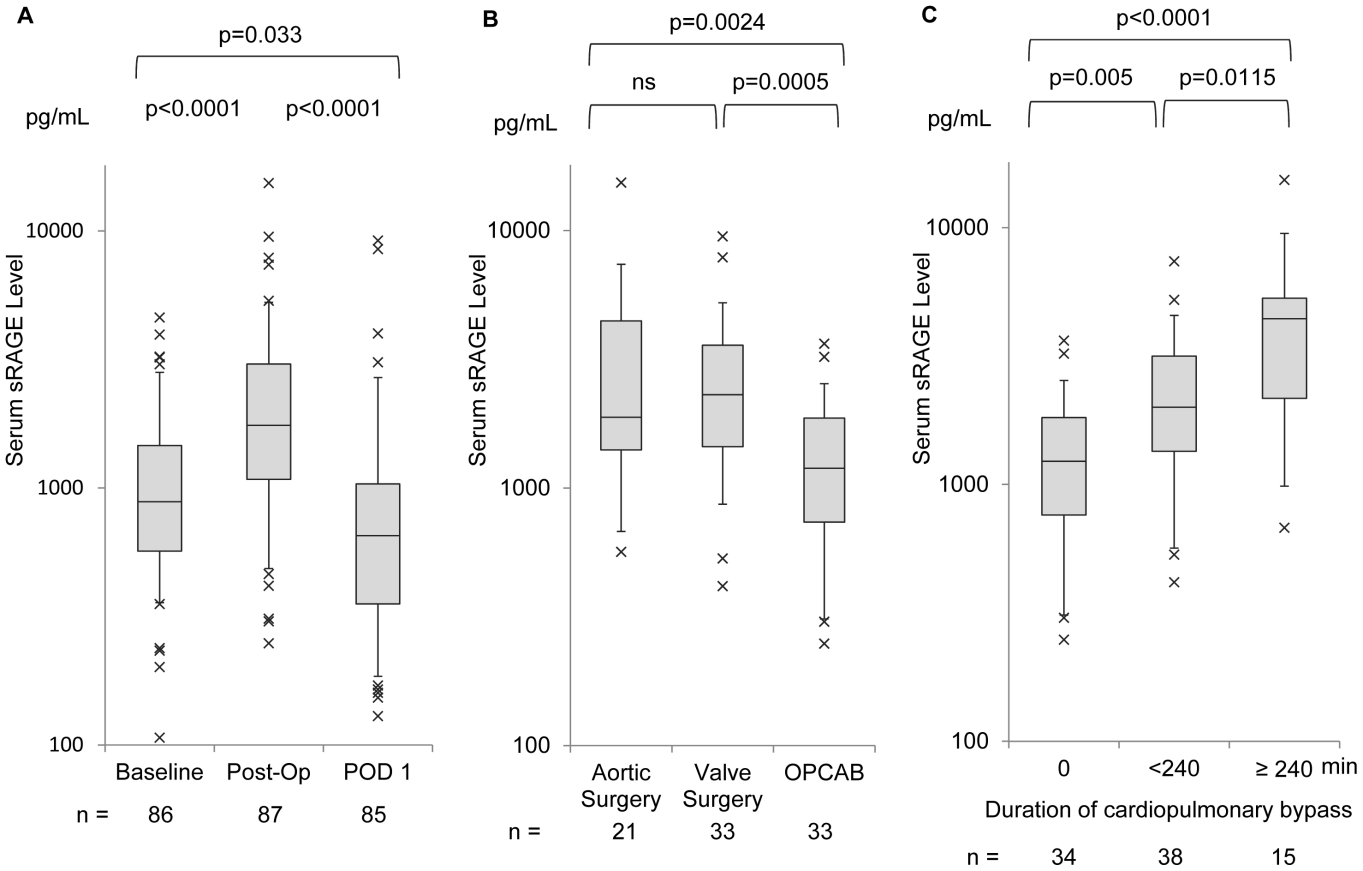
Serum levels of the soluble isoform of the receptor for advanced glycation end products (sRAGE) in the perioperative period. (A) Perioperative change in the levels of sRAGE in the entire study cohort. Serum levels of sRAGE were measured at three points: 1) after induction of anesthesia (Baseline); 2) at admission to the intensive care unit immediately after the operation (Post-Op); and 3) post-operative day 1 (POD 1); (B) Differences in Post-Op levels of sRAGE according to the type of operation. The operations were categorized as: 1) aortic surgery (*n* = 21); 2) valve surgery (*n = *33); and 3) off -pump coronary artery bypass grafting (OPCAB, *n* = 33); (C) Relationship between Post-Op sRAGE levels and duration of cardiopulmonary bypass. Patients were divided into three groups categorized by duration of cardiopulmonary bypass: 0 min; between 0 and 240 min; and ≥240 min. In panel B and C, Post-Op values were selected for comparison. Box-whisker plots show the 5th, 25th, 50th, 75th, and 95th percentile in each study condition. *p*-values for the three-group comparison were calculated using Tukey’s test. ns: not statistically significant.

The perioperative changes in the serum levels of sRAGE are shown in Figure 2A. One sample at Baseline and two samples on POD 1 were lost because of problems in sample handling. Immediately after the operation (Post-Op), serum sRAGE increased significantly from the baseline level and decreased by POD 1. In patients requiring intubation and ventilation for at least 72 h postoperatively, Post-Op sRAGE level was significantly higher than those extubated within 72h (median, 4357 pg/mL; IQR, 2335–5445 pg/mL vs. median, 1555 pg/mL; IQR, 958–2540 pg/mL, *p* = 0.0005). Figure 2B shows the differences in the serum levels of Post-Op sRAGE among the three types of operation: aortic surgery, valve surgery, and OPCAB. In the OPCAB group, Post-Op sRAGE levels were significantly lower than those in the other two groups (*p* = 0.0024 vs. the aortic surgery group; *p* = 0.0005 vs. the valve surgery group). Post-Op sRAGE levels showed a moderate correlation with the duration of CPB (*r_s_* = 0.44, *p*<0.0001), and a weak correlation with the duration of operation (*r_s_* = 0.28, *p* = 0.0081); however, there were no significant correlations between intraoperative tidal volume and Post-Op sRAGE levels. Figure 2C shows that a longer duration of CPB resulted in higher levels of Post-Op sRAGE, and sRAGE levels were significantly different among the three groups categorized by duration of CPB: 0 min; between 0 and 240 min; and ≥240 min. Clinical variables contributed to the postoperative elevation of serum levels of sRAGE was studied by multiple regression analysis, and the results are shown in Table 2. Significant correlations with Post-Op sRAGE were seen for APACHE II score (*p = *0.03), duration of operation (*p* = 0.03), and duration of CPB (*p* = 0.008); however, history of diabetes mellitus, and number of packed red cell transfusions showed no significant correlation. We also analyzed the association between Post-Op sRAGE levels and morbidity of prolonged ventilatory support (>3 days) by multiple logistic regression analysis, using these three variables (duration of operation, duration of CPB and APACHE-II score) as possible confounding factors. The results are shown in Table 3. Post-Op sRAGE levels were significantly associated with prolonged ventilatory support (*p* = 0.0009), even when correcting for APACHE-II score (*p = *0.01), duration of operation (*p = *0.009), and duration of CPB (*p = *0.005).

**Table 2:** Results of multiple regression analyses for the relationships between the serum levels of the soluble isoform of the receptor for advanced glycation end products (sRAGE) and patients' conditions.

	Coefficient.	Standard Error	P value	95% Confidence Interval
Age (year)	−0.0089	0.0093	0.34	−0.028–0.010
Sex	−0.046	0.24	0.85	−0.52–0.43
Weight (kg)	0.0049	0.0081	0.54	−0.011–0.021
Type of Surgery	−0.049	0.17	0.77	−0.38–0.28
History of diabetes mellitus	0.15	0.20	0.46	−0.25–0.54
History of smoking	−0.18	0.19	0.35	−0.56–0.20
White blood cell count (cells/mm^3^)	−0.000001	0.00002	0.97	−0.00005–0.00005
Hemoglobin (g/dL)	0.044	0.059	0.46	−0.073–0.16
Platelet count (10^4^/mm^3^)	−0.032	0.020	0.12	−0.073–0.0082
Albumin (g/dL)	−0.21	0.17	0.21	−0.54–0.12
Creatinine (mg/dL)	0.17	0.098	0.082	−0.023–0.37
PaO_2_/F_I_O_2_	−0.001	0.002	0.69	−0.001–0.002
APACHE−II score	0.076	0.0339	0.029*	0.008–0.14
Total bilirubin (mg/dL)	0.135	0.157	0.39	−0.18–0.45
Packed red cell transfusion (unit§1)	0.031	0.020	0.13	−0.010–0.072
Duration of operation (min)	−0.0020	0.0009	0.030*	−0.0038–−0.0002
Duration of cardiopulmonary bypass (min)	0.0036	0.0013	0.0075**	0.0010–0.0062
PEEP levels at ICU admission (cmH_2_O)	0.027	0.056	0.63	−0.084–0.14
Intraoperative tidal volume (mL/kg PBW§2)	0.050	0.059	0.39	−0.066–0.17
Constant	7.0	1.4	0.00	4.1–10

The serum levels of sRAGE were natural log-transformed.

§1 1 unit of packed red cells is derived from 200 mL of donated blood.

§2 PBW: predicted body weight; male PBW (kg) = 50+0.91(height (cm) –152.4), and female PBW (kg) = 45.5+0.91(height (cm) –152.4).

* Statistically significant at *p*<0.05;

** Statistically significant at *p*<0.01.

**Table 3:** Multivariable analyses of association between postoperative levels of the serum levels of the soluble isoform of the receptor for advanced glycation end products (sRAGE) and morbidity of prolonged mechanical ventilation (>3days).

Models	Odds ratio§1	95% Confidence Interval	*p*-value	
*Unadjusted*	6.9	2.2–21.9	0.0009	**
*Adjusted for*				
Duration of operation (min)	4.6	1.5–14.6	0.009	**
Duration of cardiopulmonary bypass (min)	6.7	1.8–25.1	0.005	**
APACHE-II score	5.4	1.5–20.0	0.01	*

The serum levels of sRAGE (pg/mL) were natural log-transformed.

§1 Odds ratio per 1 natural log transformed sRAGE level.

* Statistically significant at *p*<0.05;

** Statistically significant at *p*<0.01.

**Figure 3:**
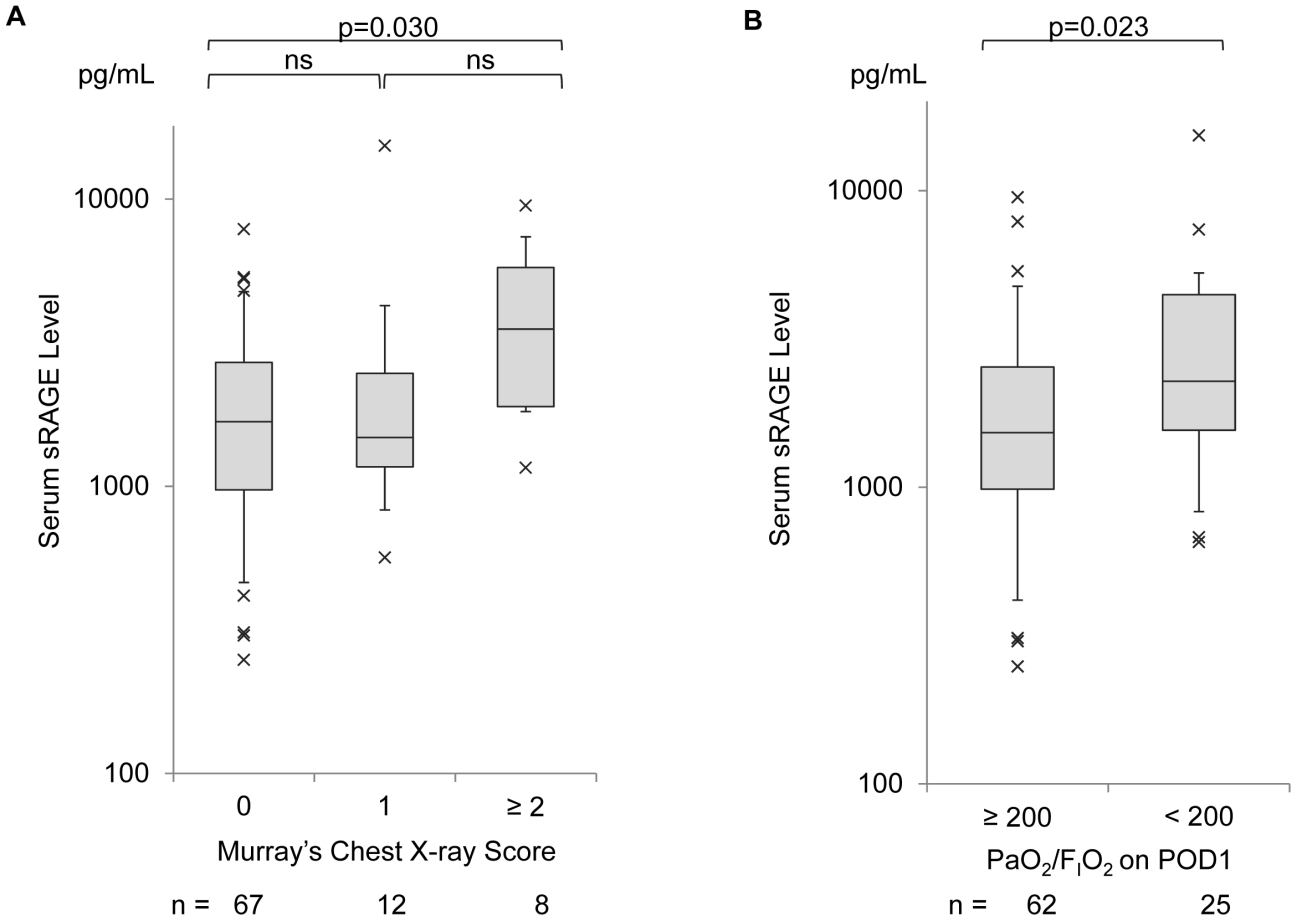
Relationship between serum levels of the soluble isoform of the receptor for advanced glycation end products (sRAGE) and the variables reflecting the condition of the lung. (A) Relationship between serum sRAGE and radiographic presentations scored by Murray’s chest X-ray score. Box-whisker plots show the 5th, 25th, 50th, 75th, and 95th percentile in each study condition. *p*-values for the three-group comparison were calculated using Tukey’s test; (B) Relationship between serum sRAGE levels and PaO_2_/F_I_O_2_ on POD 1. Patients were divided into two groups using a cut-off value for PaO_2_/F_I_O_2_ = 200. Box-whisker plots show the 5th, 25th, 50th, 75th, and 95th percentile in each study condition. The *p*-value was calculated using the Mann-Whitney U-test.

The relationship between Post-Op sRAGE levels and radiographic presentation is demonstrated in Figure 3A. Patients with the worst chest radiograph score (≥2) during the first postoperative week showed significantly higher levels of postoperative sRAGE than those with a chest radiograph score = 0 in the same postoperative period (*p* = 0.030). On POD 1, PaO_2_/F_I_O_2_ was significantly lower than the values measured immediately after operation (median, 260; IQR, 194–342 on POD 1 vs. median, 363; IQR 249–428; p = 0.0002). When patients were dichotomized by the PaO_2_/F_I_O_2_ on POD 1 with a cut-off value of 200, patients whose PaO_2_/F_I_O_2_ was <200 (*n* = 25) showed significantly higher levels of serum sRAGE than those with PaO_2_/F_I_O_2_≥200 (*n* = 62) (*p* = 0.023) (Figure 3B).

To study correlations with perioperative inflammatory reactions or endothelial injury, the correlation with interleukin-8 (median, 48 pg/mL; IQR, 28–93 pg/mL) and vWF antigen (median, 106%; IQR, 74–165%) at Post-Op were analyzed using Spearman’s correlation analysis. Post-Op sRAGE levels showed a weak positive correlation with interleukin-8 (*r_s_* = 0.28, *p* = 0.0089) and a weak negative correlation with vWF antigen (*r_s_* = −0.36, *p* = 0.0006). The results of multiple comparisons among the three surgical subgroups showed that interleukin-8 levels in the aortic surgery group (median, 100 pg/mL; IQR, 32–196 pg/mL) tended to be higher than the valve surgery group (median, 49 pg/mL; IQR, 31–79 pg/mL) with near significance (*p* = 0.065), and significantly higher than the OPCAB group (median, 35 pg/mL; IQR, 20–57 pg/mL) (*p* = 0.001). However, there were no significant differences in interleukin-8 between the valve surgery group and the OPCAB group (*p* = 0.23).

To study whether postoperative levels of sRAGE were affected by the presence of cardiac failure in our study cohort, we measured NT-proBNP as a biomarker for heart failure. The median value of Post-Op NT-proBNP level was 178 pg/mL (IQR, 81–628 pg/mL) in the total study cohort, and there were no significant correlations between NT-proBNP and Post-Op sRAGE levels (*r_s_* = 0.14, *p* = 0.20).

### Prediction of Postoperative Respiratory Failure based on the Serum Levels of sRAGE

**Figure 4:**
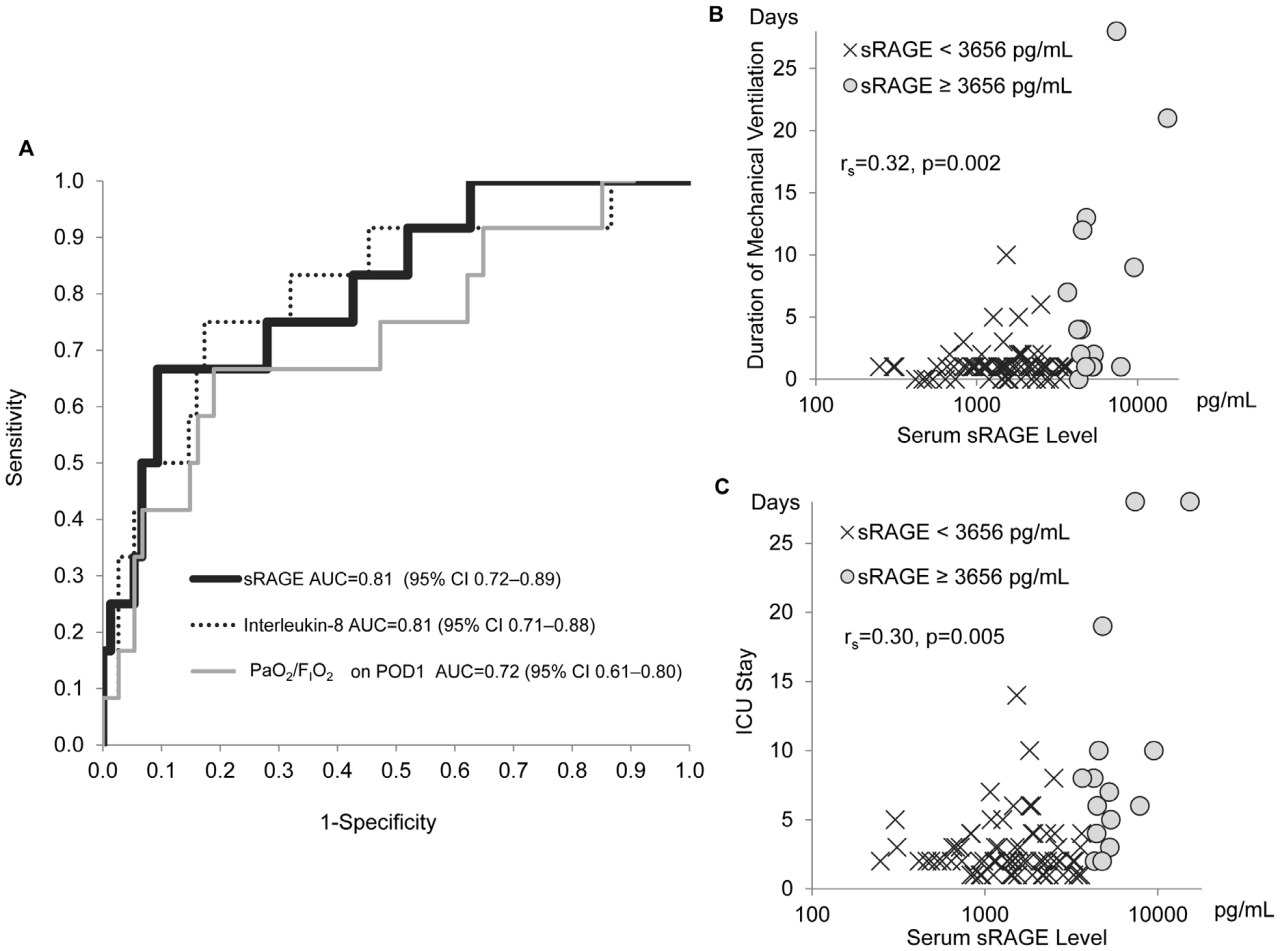
Predictive performance of the post-operative levels of the soluble isoform of the receptor for advanced glycation end products (sRAGE) for postoperative respiratory failure and prolongation of stay in the intensive care unit (ICU). (A) Receiver operating characteristic (ROC) curve analysis for predicting performance of Post-Op levels of biomarkers (sRAGE and interleukin-8) and PaO_2_/F_I_O_2_ on POD 1. ROC curves for sRAGE, interleukin-8, and PaO_2_/F_I_O_2_ are shown with their area under the curve (AUC) calculations; (B) Relationship between serum levels of sRAGE and duration of mechanical ventilation; (C) Relationship between the serum levels of sRAGE and the length of stay in the ICU. In graph B and C, patients were dichotomized using the cut-off value of 3656 pg/mL, which was calculated from the ROC curve analysis shown in graph A. Patients with a high level of sRAGE (≥3656 pg/mL) showed a significantly longer duration of mechanical ventilation (*p* = 0.0001) and a longer stay in the ICU (*p*<0.0001) than those with low sRAGE (<3656 pg/mL) (Mann-Whitney U-test). Spearman’s correlation coefficients (*r_s_*) are shown with *p* values.

We tested the potential for the post-operative levels of sRAGE to predict postoperative respiratory failure and prolongation of stay in the ICU. Figure 4A shows the receiver operating characteristic (ROC) curve for the prediction of respiratory failure, in which the area under the curve (AUC) was 0.81 (95% confidence interval (CI): 0.72–0.89). We also calculated ROC for Post-Op interleukin-8 (AUC, 0.81 with CI 0.71–0.88), and PaO_2_/F_I_O_2_ on POD 1(AUC, 0.72 with CI 0.61–0.80) (Figure 4A). Although the AUC for PaO_2_/F_I_O_2_ on POD 1 was lower than the AUC for sRAGE, the difference was not statistically significant. The optimal cut-off point for sRAGE for the prediction of respiratory failure was 3656 pg/mL, with sensitivity and specificity of 62% and 91%, respectively. When patients were divided into the two groups using this cut-off point for sRAGE, the high sRAGE group (sRAGE ≥3656 pg/mL) showed a significantly longer duration of mechanical ventilation (median, 4 days; IQR, 1–11 days vs. median, 1 day; IQR, 1–1 day, *p* = 0.0001); a longer duration of ICU stay (median, 7 days; IQR, 5–10 days vs. median, 2 days; IQR, 2–3 days, *p*<0.0001); and a lower PaO_2_/F_I_O_2_ on POD 1 (median, 178; IQR, 150–322 vs. median, 270; IQR, 205–354, *p* = 0.017).

## Discussion

RAGE is abundantly expressed in alveolar type I epithelium in the lung, and its soluble isoform (sRAGE) is released into the plasma when lung tissue is injured [12], [22]–[24]. In a study of patients with severe trauma at high risk for acute lung injury, sRAGE was one of four biomarkers with the highest diagnostic performance in a panel of 21 biomarkers for distinguishing patients with acute lung injury [25]. Furthermore, Calfee and colleagues showed that sRAGE was a marker of worse clinical outcomes in patients ventilated with higher tidal volumes in a study of 676 patients from the ARDS net trial for low tidal volume ventilation [26]. The precise mechanism of its release into plasma has not been elucidated; however, animal studies suggested that sRAGE was shed from alveolar type I epithelial cells by proteases [27], [28].

In our study, serum levels of sRAGE elevated immediately after surgery. Patients with Post-Op sRAGE ≥3656 pg/mL showed significantly lower PaO_2_/F_I_O_2_ on POD 1 than those with sRAGE <3656 pg/mL. These results are consistent with the hypothesis that sRAGE reflects postoperative lung dysfunction. Christie and colleagues studied patients undergoing lung transplantation, and demonstrated that postoperative elevation of sRAGE levels was related to primary graft dysfunction, and sRAGE levels peaked immediately after the operation [15]. Our results are consistent with this report, and Post-Op sRAGE levels demonstrated a good discriminating performance for prolonged mechanical ventilation. The predictive performance for prolonged mechanical ventilation tended to be better than postoperative PaO_2_/F_I_O_2_, probably because some surgically-induced responses and the effects of perioperative mechanical ventilation continued in the postoperative period, and the maximum reduction in PaO_2_/F_I_O_2_ might be observed later than POD 1.

Postoperative changes in the level of sRAGE were less in patients in the OPCAB group than in patients undergoing surgery with CPB (Figure 2B), and Post-Op sRAGE levels showed a moderate positive correlation with the duration of CPB. Multivariate analysis also showed a significant correlation between these two variables. Previous studies showed that insufficient bronchial artery blood flow during CPB could result in lung tissue ischemia and pulmonary lactate release, which correlated with prolonged mechanical ventilation [29], [30]. Also, CPB causes activation of the complement system and the release of various pro-inflammatory cytokines, which results in post-CPB lung dysfunction and systemic inflammation. Extracorporeal circulation increased the level of sRAGE in patients undergoing lung transplantation [15] and elective on-pump coronary artery bypass surgery [31]. Our results for sRAGE were consistent with these reports, and suggested that sRAGE was sensitive to lung dysfunction caused by CPB. Interestingly, our results showed that there were no significant differences in Post-Op sRAGE between the aortic surgery and the valve surgery groups, whereas Post-Op interleukin-8 levels in the aortic surgery group tended to be higher than in the valve surgery group with near statistical significance. This might reflect more transfusions and a longer duration of operation in the aortic surgery group, a possible additive factor for elevation of interleukin-8. In other words, Post-Op sRAGE elevation might be a more specific reaction reflecting post-CPB lung dysfunction.

We eliminated patients with pulmonary artery occlusion pressure >18 mm Hg to exclude high filling pressure-induced pulmonary edema, because cardiogenic pulmonary edema could be a confounding factor for prolonged mechanical ventilation. Although PAOP was not monitored in 25 patients, the Post-Op NT-proBNP levels in these patients were significantly lower than those in patients with PAOP monitoring. Furthermore, the Post-Op NT-proBNP in the total current study cohort was within the range previously reported from patients with acute respiratory distress syndrome [32] or transfusion-related acute lung injury [33], and Post-Op NT-proBNP levels showed no significant correlation with Post-Op sRAGE levels. Although several previous studies demonstrated a positive correlation between the severity of heart failure and sRAGE levels [34], [35], the postoperative elevation of sRAGE in our study cohort could not be explained by the mechanisms related to heart failure or cardiogenic pulmonary edema.

Previous reports have the following criticisms for the use of sRAGE as a biomarker for perioperative lung injury: First, plasma or serum levels of sRAGE begin to decrease in the postoperative period, and there is a disparity between the sRAGE levels and clinical symptoms [31], [36]. Our results also demonstrated that sRAGE levels decreased by POD 1 in almost all of the cases, regardless of their outcome. As a possible explanation, sRAGE could be released by proteolysis in the lung, induced by surgical stress and inflammation, peaking at surgery. Therefore, sRAGE could indicate the severity of alveolar epithelial damage suffered from various intraoperative insults, especially from CPB, rather than reflect a real-time change in lung epithelial condition postoperatively. Second, previous reports stated that sRAGE might not be a lung-specific marker, because extrapulmonary sources of sRAGE could affect the plasma or serum levels of this biomarker [36], [37]. However, our results showed only a weak positive correlation between serum sRAGE levels and interleukin-8. As discussed above, this weak correlation could be related to the finding that interleukin-8 tended to be more affected by systemic stress from surgery (e.g., duration of operation and numbers of transfusion) than sRAGE, causing a disparity in the pattern of elevation of the serum levels. Another explanation is that our results could reflect a small contribution by the release of sRAGE from inflammatory cells, although we could not determine the exact source of the serum sRAGE in our study design. Interestingly, in our results, Post-Op sRAGE showed a weak negative correlation with the levels of vWF antigen, a biomarker for endothelial injury. This result could be explained by the hypothesis that sRAGE might at least partially ameliorate endothelial injury by binding its pro-inflammatory ligands and function as a decoy receptor, although this endothelial anti-inflammatory effect may have no effect on the prognosis of respiratory failure. Nevertheless, this negative correlation between sRAGE and vWF antigen suggests that endothelial injury might not be a major factor in determining postoperative serum level of sRAGE in our study cohort.

Our study has limitations. First, the severity of lung injury was evaluated by one attending physician using chest radiographs, which have limited sensitivity and reproducibility for detecting lung injury. Chest computed tomography (CT) might be a better choice and a recent study demonstrated a positive correlation between CT score and plasma sRAGE levels [38]. Furthermore, our previous reports showed bronchoalveolar lavage (BAL) fluid was more sensitive for detecting local damage to lung tissue, because sRAGE concentration is higher in BAL than in blood when epithelial injury caused shedding of sRAGE into the alveolar space. Therefore, sRAGE levels in BAL could correlate better with the variables related to respiratory failure. However, this study was designed for routine clinical practices, so we included neither chest CT scans nor BAL. Determination of sRAGE levels in BAL and chest CT analysis are considerations for future studies. Second, intraoperative tidal volume was relatively high. Although the association between Post-Op sRAGE levels and intraoperative tidal volume was not significant (*r_s_* = 0.12, *p* = 0.27), high tidal volume settings might aggravate lung injury, and small tidal volume settings could result in smaller increases in postoperative sRAGE levels and lower morbidity of postoperative respiratory failure. Finally, the relatively small number of patients with postoperative respiratory failure limits the strength of the statistical power, and the size of the total study cohort was not sufficiently large to carry out a validation cohort. Large-scale multicenter studies are needed to confirm the hypotheses established from our results.

In summary, we studied the perioperative change in the serum levels of sRAGE over time and found that it elevated immediately after surgery. The range of elevation was associated with morbidity of respiratory failure after cardiac surgery, and CPB-related lung dysfunction might be a key mechanism for postoperative sRAGE elevation. Early postoperative levels of sRAGE may have predictive performance for respiratory failure after cardiac surgery; however, these hypotheses require validation in large-scale multicenter studies.
